# Combined Subcutaneous-Intranasal Immunization With Epitope-Based Antigens Elicits Binding and Neutralizing Antibody Responses in Serum and Mucosae Against PRRSV-2 and SARS-CoV-2

**DOI:** 10.3389/fimmu.2022.848054

**Published:** 2022-03-31

**Authors:** Mario Fragoso-Saavedra, Carmen Ramírez-Estudillo, Diana L. Peláez-González, Jorge O. Ramos-Flores, Gustavo Torres-Franco, Leandro Núñez-Muñoz, Gabriel Marcelino-Pérez, María G. Segura-Covarrubias, Rogelio González-González, Roberto Ruiz-Medrano, Beatriz Xoconostle-Cázares, Amanda Gayosso-Vázquez, Silvia Reyes-Maya, Vianey Ramírez-Andoney, Rogelio A. Alonso-Morales, Marco A. Vega-López

**Affiliations:** ^1^ Laboratorio de Inmunobiología de las Mucosas, Departamento de Infectómica y Patogénesis Molecular, Centro de Investigación y de Estudios Avanzados del Instituto Politécnico Nacional, Ciudad de México, Mexico; ^2^ Unidad de Producción y Experimentación de Animales de Laboratorio, Centro de Investigación y de Estudios Avanzados del Instituto Politécnico Nacional, Ciudad de México, Mexico; ^3^ Laboratorio de Biología Molecular de Plantas, Departamento de Biotecnología y Bioingeniería, Centro de Investigación y de Estudios Avanzados del Instituto Politécnico Nacional, Ciudad de México, Mexico; ^4^ Department of Physiology and Biophysics, School of Medicine, Case Western Reserve University, Cleveland, OH, United States; ^5^ Laboratorio de Genética Molecular, Departamento de Genética y Bioestadística, Facultad de Medicina Veterinaria y Zootecnia, Universidad Nacional Autónoma de México, Ciudad de México, Mexico

**Keywords:** Mucosal immunization, epitope-based antigens, mucosal antibodies, recombinant proteins, recombinant baculovirus, emerging and re-emerging viruses

## Abstract

New vaccine design approaches, platforms, and immunization strategies might foster antiviral mucosal effector and memory responses to reduce asymptomatic infection and transmission in vaccinated individuals. Here, we investigated a combined parenteral and mucosal immunization scheme to induce local and serum antibody responses, employing the epitope-based antigens 3BT and NG19m. These antigens target the important emerging and re-emerging viruses PRRSV-2 and SARS-CoV-2, respectively. We assessed two versions of the 3BT protein, which contains conserved epitopes from the GP5 envelope protein of PRRSV-2: soluble and expressed by the recombinant baculovirus BacDual-3BT. On the other hand, NG19m, comprising the receptor-binding motif of the S protein of SARS-CoV-2, was evaluated as a soluble recombinant protein only. Vietnamese mini-pigs were immunized employing different inoculation routes: subcutaneous, intranasal, or a combination of both (s.c.-i.n.). Animals produced antigen-binding and neut1ralizing antibodies in serum and mucosal fluids, with varying patterns of concentration and activity, depending on the antigen and the immunization schedule. Soluble 3BT was a potent immunogen to elicit binding and neutralizing antibodies in serum, nasal mucus, and vaginal swabs. The vectored immunogen BacDual-3BT induced binding antibodies in serum and mucosae, but PRRSV-2 neutralizing activity was found in nasal mucus exclusively when administered intranasally. NG19m promoted serum and mucosal binding antibodies, which showed differing neutralizing activity. Only serum samples from subcutaneously immunized animals inhibited RBD-ACE2 interaction, while mini-pigs inoculated intranasally or *via* the combined s.c.-i.n. scheme produced subtle neutralizing humoral responses in the upper and lower respiratory mucosae. Our results show that intranasal immunization, alone or combined with subcutaneous delivery of epitope-based antigens, generates local and systemic binding and neutralizing antibodies. Further investigation is needed to evaluate the capability of the induced responses to prevent infection and reduce transmission.

## Introduction

Almost 90% of medical and veterinary pathogens of concern infect the respiratory, enteric, and genital mucosae (1, 2). In this category, most emerging and re-emerging viruses can be found (3, 4). Such pathogens can provoke new infections in previously unaffected geographical areas or cyclical outbreaks with varying transmissibility or pathogenicity in endemic regions (4, 5). Porcine reproductive and respiratory syndrome virus (PRRSV) and severe acute respiratory syndrome coronavirus 2 (SARS-CoV-2) are two prominent examples of emerging and re-emerging viruses that infect mucosal barriers (6, 7). PRRSV is the most significant re-emerging pathogen in the pig industry; it causes a vast economic and epidemiological impact globally, being PRRSV-2 the most prevalent species in the Americas and Asia (8, 9). PRRSV-2 access to their hosts (domestic pigs) *via* respiratory, oral, and genital mucosae and replicates in monocytes and macrophages throughout the body (10, 11). SARS-CoV-2, the etiological agent of coronavirus disease 2019 (COVID-19), infects mainly the human respiratory tract (6). In both cases, the local infection can disseminate and cause a multisystemic disease: porcine reproductive and respiratory syndrome (PRRS) and severe COVID-19, respectively (12, 13). In this context, available vaccines, administered as intramuscular injections, are either suboptimal to prevent infection and disease, as in the case of PRRSV-2, or do not entirely prevent virus transmission, as evidenced in the current COVID19 pandemic (14–17). Consequently, and more than ever, an urgent need to develop effective mucosal vaccines against PRRSV-2, SARS-CoV-2, and other emerging and re-emerging viruses is compelling (18).

Mucosally administered vaccines have the potential value of preventing the establishment of infections in the first place and avoiding pathogen transmission (19, 20). Thus, the required effector immune responses include the local release of neutralizing antibodies (nAbs), especially secretory IgA, and the generation of tissue-resident memory T and B cells in mucosal surfaces and draining lymph nodes (21–24). For this to occur, the delivered antigen must breach the local physical barriers and provide the right inflammatory cues to avoid tolerogenic immune responses (25, 26). Therefore, it is not just the mucosal administration but also the antigen’s physicochemical and biological properties that influence the potency and quality of the immune responses generated. Accordingly, various experimental mucosal immunization platforms and adjuvants have been studied, including mucoadhesives, liposomes, virus-like particles (VLPs), and viral vectors (2, 27).

Viral vectors are highly immunogenic, which is both an advantage and a limitation, as the cellular and humoral immune responses elicited target the antigen of interest and the vectors themselves (28, 29). Nevertheless, viral vectors are effective immunization platforms, particularly replication-defective adenoviruses, as demonstrated in the ongoing COVID-19 pandemic (19, 20). However, preexistent immunity against some adenovirus serotypes might diminish their gene delivery capacity in humans and animals (30). An alternative vector is the baculovirus *Autographa californica* multiple nucleopolyhedrovirus (AcMNPV), which only infects insects in nature (31). Besides, AcMNPV is an efficient vector for intranasal immunization in experimental settings, given the abundant CpG oligodeoxynucleotide content in its genome, among other immunogenic properties (32).

Challenges in the vaccinology field of emerging and re-emerging viruses remain ahead of the development of mucosal vaccines and delivery platforms. Concerning PRRSV-2, difficulties include its high mutational rate, the immunomodulatory disease provoked, the virus persistence in secondary lymphoid tissues, and the documented reversion to virulence of live-attenuated virus vaccine strains (33–35). Additionally, glycosylation of PRRSV-2 proteins and the presence of immunodominant hypervariable decoy epitopes, as occurs in the GP5 protein, results in humoral immune responses that do not confer protection against heterologous strains (36, 37). Concerning SARS-CoV-2, its high transmissibility and widespread infection in the human population have led to the emergence of variants of concern (VOCs) (38). Each VOC has different mutations across its genome, including the gene coding for the spike (S) protein employed in the current commercially-available vaccines, affecting the potency of vaccine-elicited nAbs (39, 40). Consequently, the transmission-blocking efficacy of current COVID-19 vaccines against circulating VOCs is lower than anticipated (41–43). In this context, epitope-based vaccine design is an attractive method to elicit nAbs to conserved, relevant, but non-immunodominant B-cell epitopes in emerging and re-emerging viruses (44, 45). The first step is the identification of these epitopes in pathogen proteins, either through protective antibodies isolated from infected individuals or by computational methods. Then, after designing immunogens, preclinical immunization experiments are performed in animal models (45–47).

Here, as a proof-of-concept study, we investigated the production of mucosal and serum binding and nAbs induced by epitope-based antigens against PRRSV-2 and SARS-CoV-2 using a protocol of combined parenteral-mucosal immunization. To target PRRSV-2, we designed and characterized the 3BT recombinant protein, which includes a triplet of a B-cell linear epitope and a T-cell peptide from the GP5 envelope protein. We compared the local and systemic antibody responses in mini-pigs immunized either with soluble *E. coli*-expressed 3BT or the baculoviral vector BacDual-3BT. In the SARS-CoV-2 experiment, we evaluated the non-glycosylated recombinant protein NG19m, which contains the receptor-binding motif (RBM) of the S protein (48). Our results demonstrated that immunization employing epitope-based antigens elicits mucosal antibodies, either utilizing a combined subcutaneous-intranasal delivery for soluble proteins or *via* intranasal instillation of the baculoviral vector. Such local immunity might prevent infection, disease and reduce transmission of emerging and re-emerging viruses such as PRRSV-2 and SARS-CoV-2.

## Materials and Methods

### Ethics Statement

All animal procedures in this study were performed following a protocol reviewed and approved by the Institutional Animal Care and Use Committee (0315–21), following the Mexican Official Norm NOM-062-ZOO-1999.

### 3BT Epitope-Based Antigen Design

The 3BT epitope-based antigen was designed from the GP5 protein of PRRSV-2 (strain VR-2332, accession no. U87392) (**Supplementary Figure 1A**). First, the synthetic *64-3bt-gs* gene was constructed to express the 3BT-GS protein using the baculovirus expression system (**Supplementary Figure 2A**). The consensus Kozak-L21 ribosomal binding site was included at the 5´end of the DNA sequence (49). To direct 3BT-GS into the membrane of BacDual-3BT virions, the signal peptide (sp) of GP64 protein from AcMNPV was added after Kozak-L21 (50). The epitope B of GP5 (SGDSSSHLQLIYNLTLCELSGTD) was added downstream of spGP64, arranged in a triplet using the flexible linker GSAGSAAGSGEF between each repeat (51). The MHC-II restricted peptides T2 of GP5 (KGRLYRWRSPVIIEKAA) and the universal Pan DR T helper epitope (PADRE, AKFVAAWTLKAAA) were incorporated into 3BT after the triplet of epitope B (52, 53). To anchor 3BT into the lipid bilayer of virions of BacDual-3BT, amino acids 421 to 511 of the stem of VSV-G (GS) (Indiana VSV strain, accession no. J02428.1), plus a six His-Tag at its C-terminal, were placed after the MHC-II restricted peptides (**Supplementary Figure 2B**) (50). All DNA and amino acid sequences were visualized and edited using SnapGene^®^ software v5.2.4 (GSL Biotech; available at snapgene.com).

### Bioinformatic Analyses

**Figure 1:**
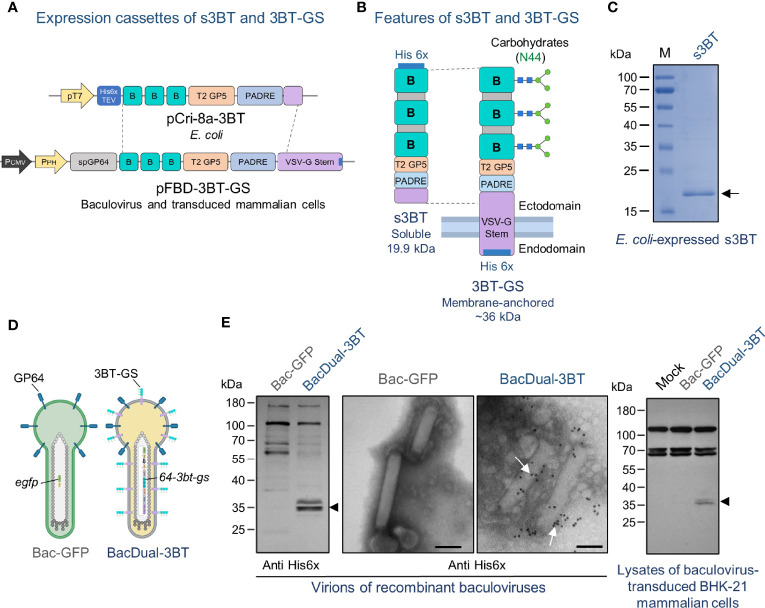
The 3BT epitope-based antigen is expressed in the soluble form by *E. coli*, displayed on the membrane of the baculovirus BacDual-3BT, and produced in transduced mammalian cells. **(A)** Constructs were obtained to express s3BT in bacteria and 3BT-GS in baculovirus. In the vector pCri-8a, s3BT expression was driven by the T7 promoter. For expression of 3BT-GS on the baculoviral envelope and in mammalian cells, the polyhedrin promoter (PPH) and the cytomegalovirus promoter (PCMV) were cloned into pFastBac™ Dual. TEV, Tobacco Etch Virus protease cleavage site; sp6P64, signal peptide of GP64. **(B)** A schematic of the two forms of 3BT epitope-based antigen that were evaluated in this study. GS, stem of VSV-G. **(C)** Coomassie-stained SDS-PAGE 10% gel, showing 10 µg of IMAC-purified s3BT protein expressed in *E. coli*. M, molecular weight marker. **(D)** Schematic representation of BacDual-3BT expressing 3BT anchored to its membrane *via* GS. GP64 is the main membrane protein of the baculovirus AcMNPV, as shown in Bac-GFP. **(E)** Left: Immunoblotting of concentrated virions of BacDual-3BT to detect 3BT-GS protein with a polyclonal anti-His6x antibody (arrowhead). Center: Immunogold electron micrographs of concentrated virions of Bac-GFP and BacDual-3BT incubated with a polyclonal anti-His6x antibody and then with an anti-rabbit-gold conjugate. White arrows in the micrograph of BacDual-3BT indicate the presence of 3BT-GS protein on the surface of virions. Bars, 100 nm. Right: Immunoblotting of lysates of baculovirus-transduced BHK-21 cells (MOI = 200 PFU/cell) to detect 3BT-GS protein with a polyclonal anti-His6x antibody (arrowhead). Figure partially created with BioRender.com.

The soluble protein 3BT (s3BT) was obtained from 3BT-GS (**Figure 1A**). The molecular weight, theoretical isoelectric point, positive and negative charged residues, grand average of hydropathicity, and instability index of 3BT-GS and s3BT were assessed with ProtParam in the Expasy server (**Supplementary Tables 1, 2**) (54). To predict the three-dimensional structure (3D) of 3BT, the amino acid sequence was analyzed with the iterative threading assembly refinement (I-TASSER) server (55). After analysis, the model with the highest confidence score (C-score) was selected for refinement through GalaxyRefine 2 server (56). To analyze the steric limitations in the polypeptide chain, a Ramachandran plot was obtained with the Ramachandran Plot Server (**Supplementary Figure 3**) (57). The visualization of 3D structures was performed with UCSF Chimera v1.15 (58). The antigenicity and prediction of linear epitope score for 3BT were evaluated in the Immune Epitope Database and Analysis Resource (IEDB), using the default threshold values (**Supplementary Figures 1B, C** **)** (13, 59, 60).

### Donor Plasmid Construction for Bac-to-Bac™ Baculovirus Expression System

We used the Bac-to-Bac™ Baculovirus Expression System (Thermo Fisher Scientific) to obtain the recombinant baculovirus BacDual-3BT, which was designed to express the 3BT-GS protein on the baculoviral membrane and in transduced mammalian cells. A synthetic cassette containing the promoters P10, CMV, and PH in tandem, was synthesized by Biomatik (Kitchener, CA) and cloned into pFastBac™ Dual (Thermo Fisher Scientific) in the restriction sites *Xho*I and *EcoR*I. The reporter gene *egfp* was PCR-amplified from pEGFP-C1 (Clontech), ligated with pTZ57R/T (Thermo Fisher Scientific), and excised with *KpnI* and *SphI* for subcloning into pFastBac™ Dual. The chimeric intron from the human β-globin and immunoglobulin heavy chain genes was PCR-amplified from the plasmid pCI-neo (Promega Corporation), ligated with pTZ57R/T, excised with *Not*I and *Hind*III, and cloned into pFastBac™ Dual, obtaining the vector pFBD-EGFP. To create the final expression vector pFBD-3BT-GS, the synthetic *64-3bt-gs* gene was cloned into pFBD-EGFP in *Sac*I and *BamH*I (**Supplementary Figure 4**). The clones were analyzed by endpoint PCR, restriction digestion, and Sanger sequencing.

### Soluble 3BT Expression in *E. coli*

To obtain the plasmid for the expression of soluble 3BT (s3BT) in *E. coli*, the pCri-8a expression vector was used (61). The DNA sequence coding for the ectodomain of 3BT-GS (without the spGP64) was PCR-amplified from the synthetic cassette 64-*3bt-gs* (**Figure 1A**). The PCR product was cloned into pCR^®^8/GW/TOPO^®^ (Thermo Fisher Scientific), excised with *Not*I and *Hind*III, and cloned into pCri-8a to obtain pCri-8a-3BT. The clones were analyzed by endpoint PCR, restriction digestion, and Sanger sequencing. For protein expression, pCri-8a-3BT transformed *E. coli* BL21 DE3 bacteria were grown in 4 L of LB culture medium with kanamycin. Induction was carried out with 1 mM IPTG (Isopropyl β-d-1-thiogalactopyranoside, BioRad) for 16 h at 19°C. Cultures were centrifugated, and the pellets were resuspended with lysis buffer (30 mM Tris, 500 mM NaCl, 20 mM imidazole, 1 mM phenylmethylsulfonyl fluoride, pH 8.0), sonicated, and further centrifugated. The soluble protein fraction was purified by immobilized metal affinity chromatography (IMAC) with Ni-NTA resin, according to the manufacturer´s instructions (HisTrap, Amersham Biosciences). Then, s3BT was recovered in five volumes of lysis buffer with imidazole 500 mM, dialyzed overnight against PBS at 4°C, and its purity was assessed by Coomassie staining after 10% sodium dodecyl sulfate-polyacrylamide gel electrophoresis (SDS-PAGE). The protein was aliquoted and preserved with cOmplete mini protease inhibitor (Roche) at −80°C until used.

### NG19m Antigen

NG19m is a recombinant *E. coli*-expressed epitope-based antigen against SARS-CoV-2. Its design, expression, purification, and immunogenicity were previously reported (48).

### Cell Culture

Sf9 cells (ATCC^®^ CRL-1711™) were grown in Sf-900™ II serum-free medium (Gibco™) at 27°C in suspension. MARC-145 (ATCC^®^ CRL-12231™) cells were maintained in Dulbecco’s Modified Eagle’s Medium (DMEM, Gibco™), while BHK-21 cells (ATCC^®^ CCL-10™) were grown in Eagle’s Minimum Essential Medium (EMEM, Gibco™). DMEM and EMEM were supplemented with 10% fetal bovine serum (FBS, HyClone, Thermo Fisher Scientific) and penicillin (100 U/mL)/streptomycin (100 μg/mL)/amphotericin B (0.25 μg/mL) (Gibco™). MARC-145 and BHK-21 cells were kept at 37°C in a humidified incubator with 5% CO_2_.

### Recombinant Baculovirus Production and Propagation

Competent DH10Bac™ *E. coli* cells were transformed with the donor plasmid pFBD-3BT-GS, following the manufacturer’s recommendations. Each recombinant bacmid DNA was purified by in-house minipreps (62) for transfection into Sf9 cells, using *Trans*IT^®^-Insect Transfection Reagent (Mirus Bio LLC). Passage 0 (P0) of BacDual-3BT was harvested and titrated as previously described (63). To obtain high titer P2 and P3 of Bac-GFP and BacDual-3BT, Sf9 cells at 1.2 x 10^6^ cells/mL were infected with a multiplicity of infection (MOI) of 0.1 PFU/cell. At 80 h post-infection, baculoviral supernatants were harvested (64). To reach Bac-GFP and BacDual-3BT titers of 1 x 10^9^ PFU/mL, utilized for immunization, supernatants were centrifugated at 14,000 x *g* 90 min at 4°C. Supernatants were removed, and viral pellets were soaked with sterile/endotoxin-free PBS overnight at 4°C. Baculoviral pellets were gently resuspended, titrated, aliquoted, and stored protected from light at 4°C (64).

### Immunogold-Electron Microscopy

Immunogold-electron microscopy was carried out with samples of concentrated virions of Bac-GFP and BacDual-3BT as described elsewhere (65, 66). A rabbit polyclonal anti-His 6x primary antibody (1:10) (Bethyl Laboratories Inc.) and a 10 nm colloidal gold-conjugated goat anti-rabbit IgG secondary antibody (1:20) (Sigma-Aldrich) were utilized. Antibodies were diluted in 10% normal goat serum (Sigma-Aldrich) in 1x PBS.

### PNGase F Treatment of Virions of BacDual-3BT

Concentrated virions of BacDual-3BT (10 µg) were incubated at 37°C with 2.5 µL (0.125 units) of PNGase F (Sigma-Aldrich) for 3 h. After incubation, digested virions were separated by 10% SDS-PAGE and evaluated by immunoblot, as described below.

### PRRSV-2 Propagation, Titration, Inactivation, and Ultracentrifugation

A PRRSV-2 VR-2332-derived isolate was propagated in MARC-145 cells, as previously described (67). The protocol to titrate PRRSV-2 was adapted from (67), detecting the nucleoprotein (N) by an indirect in-cell ELISA with the mAb SDOW17 (1:6,000) (Rural Technologies Inc.) and a goat anti-mouse IgG1-HRP secondary Ab (1:40,000) (Bethyl Laboratories Inc.). Antibodies were diluted in 1% BSA dissolved in 1x PBS. The optical density (O.D.) at 450 nm was measured in a microplate reader (Multiskan EX, Thermo Scientific). TCID_50_ and PFU/mL values were calculated applying the formula of Reed and Muench and the statistical relationship PFU = 0.69 x TCID_50_, respectively (63). Virions of PRRSV-2 used for immunization or ELISA were thawed overnight at 4°C, inactivated in a water bath at 56°C for 30 min, and checked for infectivity in confluent monolayers of MARC-145 cells (68). Inactive virions were concentrated by ultracentrifugation through a sucrose cushion, as described in (69). Pelleted virions were resuspended with ice-cold TE7 buffer (10 mM Tris-HCl, 1 mM EDTA, pH 7.0, with cOmplete mini protease inhibitor from Roche), quantified by Pierce™ BCA Protein Assay Kit (Thermo Fisher Scientific) as indicated by the manufacturer, aliquoted, and stored at -80°C until use.

### Transduction of Mammalian Cells

Mammalian BHK-21 cells were transduced with Bac-GFP or BacDual-3BT (MOI = 200 PFU/cell), as reported in (62). Cell lysates were obtained using NET cell-lysis buffer (20 mM Tris, 150 mM NaCl, 1% Triton X-100, 1 mM EDTA, with cOmplete mini protease inhibitor from Roche). Total protein in recovered supernatants was quantified by 2-D Quant kit (GE Healthcare), as indicated by the manufacturer, and immediately evaluated by immunoblot.

### SDS-PAGE and Immunoblot

Cell lysates and pelleted PRRSV-2 virions (20 μg), or 8 μg of concentrated virions of baculoviruses were separated by 10% SDS-PAGE, transferred to 0.45 μm nitrocellulose membranes (Bio-Rad Laboratories), and blocked for non-specific binding with 3% BSA dissolved in 1x TBS with 0.05% Tween 20 (TBS-T). Membranes were probed with rabbit polyclonal anti-His 6x primary antibody (1:15,000) (Bethyl Laboratories Inc.) or pig anti-PRRSV-2 hyperimmune sera (1:750). After TBST washing, membranes were probed with a biotinylated goat anti-rabbit IgG (1:800,000) or goat anti-pig IgG-HRP (1:15,000) (Bethyl Laboratories Inc.). Streptavidin-HRP (1:8,000) (Jackson ImmunoResearch) was utilized in membranes incubated with the biotinylated secondary antibody. All sera and antibodies were diluted in blocking buffer. Bound proteins were visualized by using Pierce™ ECL Western Blotting Substrate (Thermo Fisher Scientific) and medical X-ray general purpose blue radiographic films (Eastman Kodak Company).

### Animals and Immunization Protocols

Two or three-month-old specific pathogen-free (SPF) weaned male and female Vietnamese minipigs were employed for immunization, constituting six groups for the PRRSV-2 experiment and four groups for the SARS-CoV-2 experiment (n = 4-6/group). Animals were immunized four times at days 0, 7, 14, and 42, through either subcutaneous (s.c.) or intranasal (i.n.) routes, depending on the group. For s.c. jabs with heat-inactivated PRRSV-2 or s3BT protein (PRRSV-2 experiment), a volume containing 100 µg of the corresponding antigen was mixed with an equal volume of incomplete Freund´s adjuvant (IFA) (Sigma-Aldrich) and injected into the neck. In the SARS-CoV-2 experiment, animals were subcutaneously immunized with a volume containing 100 µg of NG19m protein mixed with an equal volume of 5 mg of aluminum hydroxide (Imject™ Alum Adjuvant, Thermo Fisher Scientific). For i.n. doses, 200 μg of the appropriate antigen without adjuvant were instilled into the nostrils. Animals immunized with recombinant baculoviruses received 5 x 10^8^ PFU in the first two doses and 1 x 10^9^ PFU in the third and fourth doses, irrespective of the inoculation route. At the end of the experiments, animals were humanely euthanized; bronchoalveolar lavages (BAL) were obtained as detailed in (70) from pigs immunized with the NG19m antigen.

### Collection of Samples and Quantitative ELISA

Blood, nasal mucus, saliva, and vaginal swabs were obtained at days 0, 14, 28, 42, and 56 of the immunization schemes. These samples were collected and processed as detailed previously (71) and stored at -20°C until analysis. Serum and mucosal antigen-binding IgG and IgA were measured by a quantitative ELISA adapted in our laboratory (45, 46).

### Microneutralization Assays

The capacity of serum IgG from s3BT and BacDual-3BT-immunized animals to bind PRRSV-2 was assessed by in-cell ELISA. Confluent MARC-145 cells grown in 96 well-plates were infected with a PRRSV-2 VR-2332-derived isolate (100 TCID_50_) for 48 h. Infected cells were fixed, permeabilized, and blocked, as described for PRRSV-2 titration (67). Serum samples collected at day 56 were twofold diluted in TBST, starting from 1:50, and added to cells. After an incubation of 1 h at room temperature, a goat anti-pig IgG-HRP secondary Ab (1:200,000) (Bethyl Laboratories Inc.) was utilized. The O.D. at 450 nm was measured in a microplate reader (Multiskan EX, Thermo Scientific). Then, the neutralization activity of sera and mucosal secretions against PRRSV-2 was evaluated as described in (72, 73), with some adjustments. Sera and mucosal secretions collected at day 56 were diluted in DMEM with penicillin (100 U/mL)/streptomycin (100 μg/mL)/amphotericin B (0.25 μg/mL), and gentamicin (50 µg/mL) (Gibco™), starting from 1:4 to 1:512. PRRSV-2 VR-2332-derived isolate (100 TCID_50_) was incubated for 1 h at 37°C with each serially diluted sample, added to confluent MARC-145 cells grown in 96 well-plates, and incubated for 2h at 37°C. Sample-virus mixes were removed, fresh DMEM with antibiotics and 5% FBS was added to cells, and plates were placed for 48 h at 37°C. To evaluate infection, the nucleoprotein of PRRSV-2 was detected by an indirect in-cell ELISA, as described for PRRSV-2 titration. The percent inhibition of virus infection at each well and ID_50_ titers were calculated as described in (72). For the SARS-CoV-2 experiment, neutralization activity of sera and mucosal secretions collected at day 56 was analyzed using the SARS-CoV-2 Surrogate Virus Neutralization Test (sVNT) (GenScript Biotech), according to the manufacturer’s recommendations; two convalescent human sera were employed as positive controls.

### Statistical Analyses

All data were analyzed and graphed in GraphPad Prism software, version 9.2.0 (GraphPad Software, San Diego, California USA, www.graphpad.com). Tests were selected according to the type of experiment and are indicated in the corresponding figures. An alpha value of 0.05 was set for all analyses.

## Results

### Bioinformatic Analyses Predict the Feasibility of the 3BT Epitope-Based Antigen to Induce Humoral Immune Responses

Epitope B is a glycosylated linear non-immunodominant B-cell epitope localized in the ectodomain of GP5 of PRRSV-2, and it is flanked by immunodominant hypervariable decoy epitopes A and C (74) (**Supplementary Figure 1A**). Previous reports indicate that epitope B is a target for nAbs and is conserved among several PRRSV-2 strains (74–77). Despite the evidence, epitope B has not been thoroughly evaluated as a subunit vaccine candidate against PRRSV-2. To investigate if epitope B could elicit nAbs against PRRSV-2, we designed the 3BT epitope-based antigen. We included a triplet of epitope B (3B) found in the PRRSV-2 VR-2332 reference strain and arranged it in tandem with two flexible linkers in between. Tandem repeats of linear B-cell epitopes might augment BCR-crosslinking, B-cell endocytosis, and presentation to CD4^+^ helper T-cells (78, 79). To enhance antibody production, we inserted two MHC-II restricted epitopes in 3BT that could improve CD4^+^ T-cell help to B cells: T-cell epitope 2, a previously characterized epitope found in the endodomain of GP5 (52), and Pan DR T helper epitope (PADRE), a universal synthetic epitope which, although designed to bind HLA-DR molecules in humans (80), it also potentiates humoral responses in pigs (81, 82). In this way, our 3BT epitope-based antigen was constituted similarly to a conjugate protein-protein vaccine against the non-immunodominant epitope B of PRRSV-2 (**Supplementary Figure 1A**).

To assess 3BT antigenicity, we subjected its amino acid sequence to bioinformatic analyses. The antigenicity of 3BT was examined using the Kolaskar and Tongaonkar antigenicity scale, which compares the physicochemical properties and the frequencies of amino acids in the target sequence with those from experimentally known linear epitopes (83). The core sequence of each epitope B in the triplet had a value of 1.13 (above the threshold default of 1.021), indicating the high antigenicity of each repeat (**Supplementary Figure 1B**). Subsequently, we obtained a three-dimensional model of 3BT with an adequate quality (**Supplementary Figure 3**) and used it to predict the linear epitope score for each copy of epitope B in 3BT (**Supplementary Figure 1C**). The score obtained was above the threshold of 0.5, indicating that each epitope B protrudes from the protein´s globular surface with high solvent accessibility and flexibility (84). Overall, these results showed that our 3BT epitope-based antigen was a strong candidate to elicit antibody responses.

### The 3BT Epitope-Based Antigen Is Adequately Produced by *E. coli*, Displayed on the Envelope of BacDual-3BT, and Expressed by Baculovirus-Transduced Mammalian Cells

In this work, we generated two versions of our 3BT epitope-based antigen: *E. coli-*expressed soluble 3BT (s3BT) and membrane-anchored 3BT-GS, either displayed by BacDual-3BT virions or produced in baculovirus-transduced mammalian cells. Theoretical physicochemical properties of s3BT and 3BT-GS are presented in **Supplementary Table 1**. To obtain the pFBD-3BT-GS plasmid for the baculovirus expression system, we first cloned the synthetic *64-3bt-gs* gene into the pFBD-EGFP vector, a modified version of pFastBac^™^ Dual (**Supplementary Figure 4** and **Supplementary Table 2**). To obtain the s3BT *E. coli-*expression vector, the DNA sequence coding for 3BT and a stretch of the ectodomain of GS was PCR-amplified from pFBD-3BT-GS and cloned into the pCri-8a plasmid (**Figure 1A** and **Supplementary Table 2**). Both s3BT and 3BT-GS proteins included a His6x tag to facilitate their detection and purification. Only 3BT-GS was glycosylated at the Asn present in each copy of epitope B, which was predicted to result in a ~36 kDa protein (**Figure 1B** and **Supplementary Figure 5A**).

After obtaining pCri-8A-3B vector, soluble 19.9 kDa His6x-tagged 3BT was expressed in *E. coli* BL21, purified by affinity chromatography, and visualized on an SDS-PAGE gel stained with Coomassie blue (**Figure 1C**).

The signal peptide of the GP64 (present in 3BT-GS) aids in the incorporation of recombinant transmembrane proteins into the baculovirus envelope (50), as represented in **Figure 1D**. To examine if the baculovirus BacDual-3BT expressed 3BT-GS, we analyzed concentrated virions by immunoblot and immunogold-electron microscopy. Using a primary anti-His6x antibody, we detected two 35-36 kDa bands in Western blot membranes, which might be two different molecular forms of the glycosylated protein (**Figure 1E**, left panel). To further test this hypothesis, concentrated virions of BacDual-3BT were treated with the enzyme PNGase F (which removes N-linked glycans), separated in an SDS-PAGE gel, and examined through immunoblot. We found a reduction in the molecular weight of one of the bands while the other remained unchanged (**Supplementary Figure 5B**). Such a finding may indicate that some of the N-linked glycans in 3BT-GS (mannose and paucimannose) were resistant to PNGase F (85).

Furthermore, electron micrographs revealed the presence of His6x-tagged 3BT-GS on the virions of BacDual-3BT (**Figure 1E**, middle panel). Also, as the *64-3bt-gs* gene carried by BacDual-3BT included the cytomegalovirus promoter (PCMV), we detected 3BT-GS protein by immunoblot in BacDual-3BT-transduced mammalian BHK-21 cells (**Figure 1E**, right panel). To examine if antibodies raised against PRRSV-2 could bind to 3BT-GS expressed on BacDual-3BT, we conducted an immunoblot of concentrated virions, using two anti-PRRSV-2 convalescent swine sera. Antibodies in each serum recognized 3BT-GS on virions of BacDual-3BT and GP5-M proteins on samples of concentrated PRRSV-2 virions (**Supplementary Figure 6**). These results demonstrated that BacDual-3BT works as a transduction vector and that displayed tandem repeats of epitope B on its surface might be conformationally similar to native epitope B in GP5.

### Mucosal Immunization With Epitope-Based Antigens: PRRSV-2 and SARS-CoV-2 Experiments

**Figure 2:**
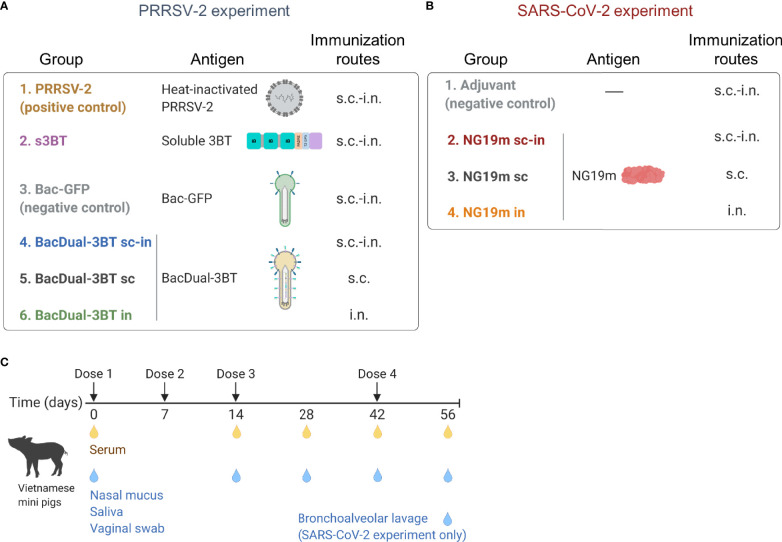
Groups of immunized animals and the immunization/sample collection schedule in PRRSV-2 and SARS-CoV-2 experiments. **(A, B)** Schematic representation of antigens and immunization routes assessed in each experiment. Groups were constituted of four (Bac-GFP) to five or six animals (rest of the groups). **(C)** Two and three-month-old, SPF weaned Vietnamese male and female mini-pigs were employed in both experiments. Timepoints of immunization and serum/mucosal sample collection are indicated. Figure created with BioRender.com.

To evaluate the serum and mucosal binding antibodies and nAbs produced after immunization with 3BT and NG19m, we conducted PRRSV-2 and SARS-CoV-2 experiments. In the PRRSV-2 experiment, we immunized Vietnamese SPF mini-pigs with either s3BT or BacDual-3BT. Regarding the SARS-CoV-2 experiment, animals were inoculated with NG19m, a previously characterized epitope-based antigen that comprises a non-glycosylated region (S371-F541) within the RBD of the S protein of SARS-CoV-2 (48). We utilized a combined subcutaneous-intranasal (s.c.-i.n.) immunization scheme by default in both experiments (**Figures 2A, B** **)**. Such a s.c.-i.n. protocol consists in the administration of the adjuvanted antigen (or BacDual-3BT without adjuvant) in two s.c. doses, followed by two i.n. instillations of the non-adjuvanted antigen, generating binding IgG and IgA in serum, nasal mucus, and saliva (71, 86, 87).

Additionally, s.c. and i.n. groups were constituted in both experiments (**Figures 2A, B** **).** Animals received their first dose of each antigen at day 0, a second dose at day 7, and two boosts at days 14 and 42 (**Figure 2C**). Serum, nasal mucus, and saliva were obtained at days 0, 14, 28, 42, and 56 (**Figure 2C**), and antigen-binding IgG and IgA concentrations were measured by ELISA. Vaginal swab samples were also evaluated for the PRRSV-2 experiment, as this barrier tissue is susceptible to infection (88). BAL samples were collected at the end of the SARS-CoV-2 experiment only, given the importance of immune response against this pathogen in the lower respiratory tract (12). In both experiments, nAbs were analyzed in the collected samples at the end of the immunization schedule (day 56).

### Combined S.C.-I.N. Immunization With Heat-Inactivated PRRSV-2 Drives the Production of Antigen-Binding IgG and IgA in Serum and Mucosal Fluids

To evaluate the dynamics of serum and mucosal PRRSV-2-binding IgG and IgA production, mini-pigs from the positive control group received 100 µg of heat-inactivated PRRSV-2 in two s.c. doses, mixed with incomplete Freund adjuvant (IFA). In the two i.n. boosts, 200 µg of the antigen without adjuvant were instilled into the nostrils. In sera, PRRSV-2-binding IgG and IgA concentrations augmented progressively with a slight ascent after the boost of day 42 (**Supplementary Figures 7A, B**). In mucosal fluids, the most abundant Ig class varied depending on the sample. In nasal mucus, PRRSV-2-binding IgA augmented after the first intranasal boost to reach ~25 ng/mL, while IgG was negligible. IgG was more abundant in saliva than IgA, and in the vaginal swab a higher concentration of IgG was detected (**Supplementary Figures 7A, B**). These results confirmed the utility of the s.c.-i.n. immunization scheme to induce antigen-binding IgG and IgA in serum and mucosal fluids.

### Combined S.C.-I.N. Immunization With s3BT Elicits High Concentrations of Antigen-Binding IgG and IgA in Serum and Mucosal Fluids

**Figure 3:**
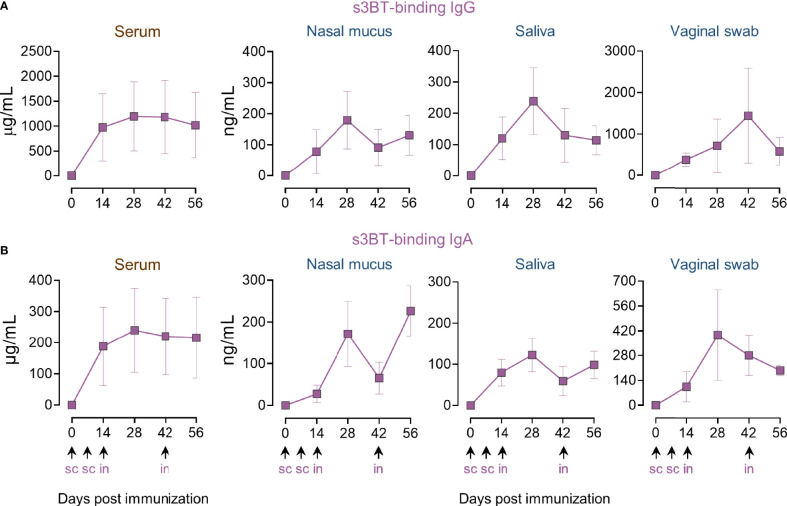
Combined s.c.-i.n. immunization with s3BT results in the production of antigen-binding IgG and IgA in serum and mucosal fluids. Animals were inoculated with 100 µg of purified s3BT protein mixed with IFA 1:1 (v/v) in the first two s.c. doses, and 200 µg of s3BT without adjuvant for intranasal immunizations. Dynamics of serum and mucosal anti-s3BT IgG **(A)** and IgA **(B)**, assayed by quantitative ELISA using s3BT. The scales are different in the vaginal swab graphs. Error bars represent SEM.

To evaluate the immunogenicity of s3BT, animals were immunized using the combined s.c.-i.n. scheme as in the PRRSV-2 group (100 µg s.c. plus 200 µg i.n.). ELISA results revealed that animals generated higher concentrations of s3BT-binding IgG and IgA in serum and mucosae in comparison to PRRSV-2-immunized pigs. In serum, the maximum anti-s3BT IgG and IgA levels were reached after day 14; for IgG, the average concentration was 1 mg/mL and 200 µg/mL for IgA (**Figures 3A, B** **)**. In nasal mucus and saliva, s3BT-binding IgG and IgA were detected at similar concentrations that fluctuated across the immunization schedule but ended at similar levels in the range of 100-250 ng/mL. An average of ~800 ng/mL of s3BT-binding IgG was quantified in vaginal swab samples, while at day 56 IgA concentration was ~200 ng/mL (**Figures 3A, B** **)**. These results indicated that s3BT is highly immunogenic and yields a robust antigen-binding antibody response in serum and mucosal fluids.

### Immunization With BacDual-3BT Induces Different Quantities of 3BT-Binding IgG and IgA in Serum and Mucosal Fluids, Depending on the Inoculation Route

**Figure 4:**
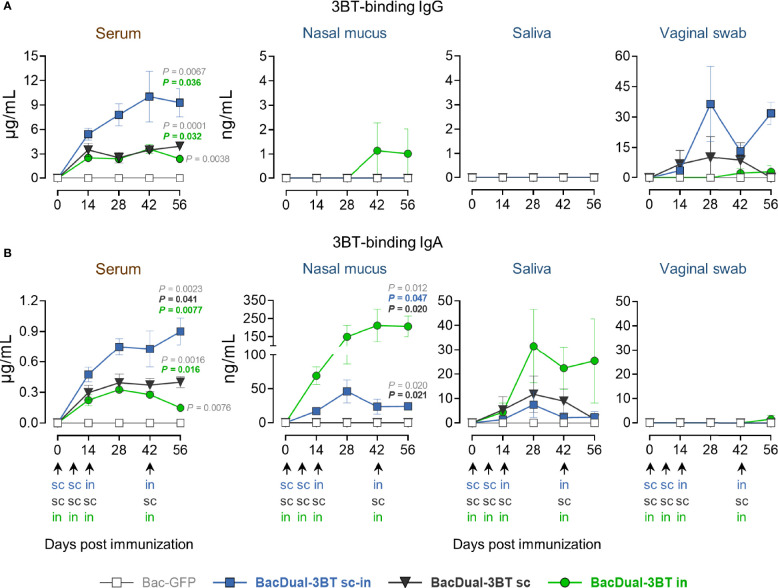
BacDual-3BT-immunized animals generated different quantities of 3BT-binding IgG and IgA in serum and mucosal fluids, depending on the inoculation route. Mini-pigs were vaccinated with 5 x 10^8^ PFU of recombinant baculoviruses in the first two doses, regardless of the route, and 1 x 10^9^ PFU in the last two doses. Kinetics of serum and mucosal 3BT-binding IgG **(A)** and IgA **(B)**, assayed by quantitative ELISA using *E. coli* expressed s3BT. Differences in the levels of antibodies among the groups were analyzed utilizing two-way ANOVA with the Geisser-Greenhouse correction, followed by Dunnett´s (comparison with the control group) and Tukey´s (comparison among the groups) multiple comparisons. Graph scales are different and error bars represent SEM. P values ≤ 0.05 are indicated for day 56 only.

Recombinant viral vectors, including the baculovirus AcMNPV, elicit robust local immune responses when administered intranasally (18, 32). To determine the best inoculation route to produce 3BT-specific antibodies in serum and mucosae, mini-pigs were immunized with virions of BacDual-3BT, either utilizing the combined s.c.-i.n. protocol, the s.c. route, or the i.n. route only (**Figure 2A**). Mini-pigs received 5 x 10^8^ PFU of BacDual-3BT in the first two doses and 1 x 10^9^ PFU in the last two inoculations. To analyze the dynamics of the production of antigen-specific antibodies, *E.* coli-expressed s3BT was employed for ELISA, as we had difficulties obtaining pure 3BT-GS from BacDual-3BT-infected insect cells. In serum, animals in the s.c.-i.n. (*P* = 0.036) and s.c. (*P* = 0.032) groups produced a significantly higher concentration of 3BT-binding IgG in comparison to the i.n. group. Animals from the s.c.-i.n. group generated a superior 3BT-binding IgA concentration in serum than the s.c. (*P* = 0.041) or i.n. (*P* = 0.0077) groups (**Figures 4A, B** **)**. IgG was absent in nasal mucus and saliva but detectable in vaginal swab samples, especially for the s.c.-i.n. group (**Figure 4A**). IgA concentration was variable in saliva and non-detected in the vaginal swab samples. However, animals immunized with BacDual-3BT through the i.n. route generated a high concentration of 3BT-binding IgA, significantly superior to the s.c.-i.n. (*P* = 0.047) and s.c. (*P* = 0.020) groups (**Figure 4B**), comparable to the s3BT-specific nasal IgA produced by s3BT-immunized animals (**Figure 3B**). Collectively, our data demonstrated that BacDual-3BT is a good immunogen to promote the generation of antigen-binding antibodies in sera when administered s.c.-i.n. or s.c. and a potent immunization vector to elicit antigen-specific IgA locally when delivered i.n. As an addendum, antibodies raised against the baculoviral vector in sera and mucosae were ten to twenty times higher in concentration than s3BT-binding IgG and IgA (**Supplementary Figure 8**), suggesting that the viral vector may not be used more than once within an immunization protocol.

### NG19m-Immunized Pigs Generate Antigen-Specific Serum and Mucosal Antibodies

**Figure 5:**
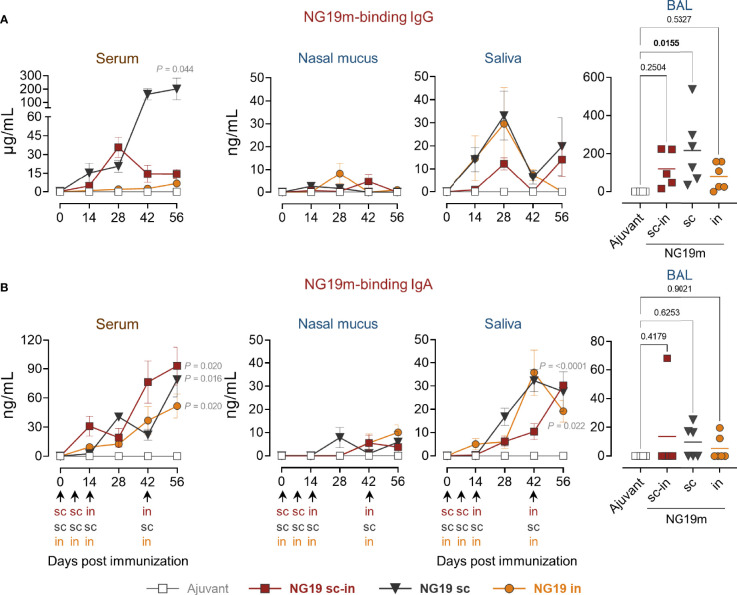
NG19m-immunized pigs generated antigen-specific serum and mucosal antibodies. Animals were immunized in the first two doses with 100 µg of NG19m protein mixed with alum 1:1 (v/v) and 200 µg of NG19m without adjuvant in the third and fourth immunizations. Kinetics of serum and mucosal NG19m-binding IgG **(A)** and IgA **(B)**, assayed by quantitative ELISA using NG19m protein. Differences in the kinetics of antibodies were analyzed utilizing two-way ANOVA with the Geisser-Greenhouse correction, followed by Dunnett´s (comparison with the control group) and Tukey´s (comparison among the groups) multiple comparisons. Ordinary one-way ANOVA followed by Tukey´s multiple comparisons was applied to evaluate differences in BAL samples. In BAL graphs the scales are different. Error bars represent SEM. In the kinetics of antibody production graphs, P values ≤ 0.05 are indicated for day 56 only.

In the SARS-CoV-2 experiment, mini-pigs were immunized with the NG19m epitope-based antigen and the humoral immune responses among the s.c.-i.n., s.c., or i.n. groups were contrasted (**Figure 2B**). Animals received 100 µg of NG19m in the first two doses using alum instead of IFA because NG19m is a COVID-19 vaccine candidate in Mexico. In the last two doses, 200 µg of NG19m were administered *via* the corresponding routes according to the groups. Mini-pigs generated NG19m-binding IgG and IgA in serum. IgG concentration was higher for the s.c. group, rounding 200 µg/mL, regarding s.c.-i.n. or i.n. groups (**Figure 5A**). NG19m-binding IgA levels in serum were similar among the groups (**Figure 5B**). In nasal mucus, IgG and IgA levels were scarcely detected, and NG19m-binding IgA in saliva rounded 20-30 ng/mL at day 56 for all the groups (**Figures 5A, B** **)**. In BAL samples, animals from the s.c. group produced higher levels of antigen-specific IgG than the s.c.-i.n. or i.n. groups (**Figure 5A**). IgA levels in BAL samples from all the groups were below 20 ng/mL on average (**Figure 5B**). These results showed that NG19m produces a better serum and mucosal humoral immune response when injected subcutaneously, but there is room for future adjustments in the applied doses to improve results.

### Soluble 3BT, BacDual-3BT, and NG19m Elicit a Variable nAb Response Against the Target Viruses in Serum and Mucosal Fluids

**Figure 6:**
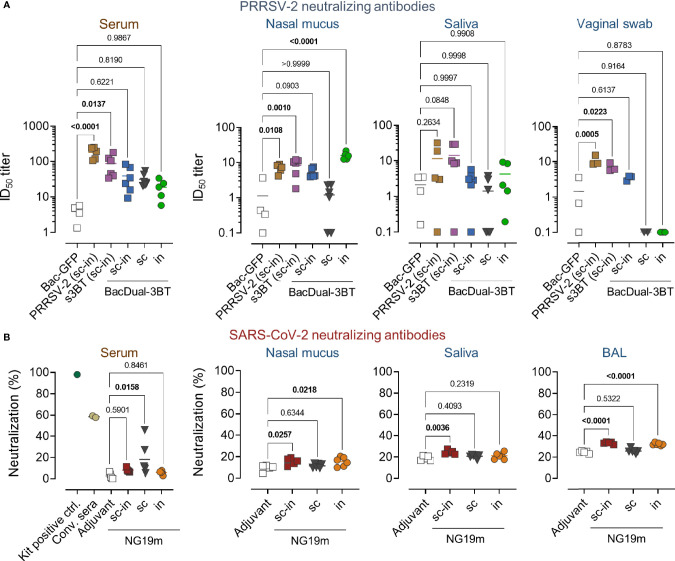
Soluble 3BT, BacDual-3BT, and NG19m elicit a variable neutralizing antibody response against the target viruses in serum and mucosal fluids. The neutralizing activity of serum and mucosal samples, collected at day 56 of the immunization scheme in PRRSV-2 and SARS-CoV-2 experiments, was assessed. **(A)** The ability of serum and mucosal samples to interfere with PRRSV-2 infection was measured *via* in-cell ELISA. MARC-145 cells grown in 96-well plates were incubated with each sample dilution-virus mixture (100 TCID_50_). The anti-N monoclonal antibody SDOW-17 was utilized to detect N protein in fixed and permeabilized cells. The half-maximal inhibitory dilution (ID_50_) is presented for each sample. Antibiotics (penicillin, streptomycin, and gentamycin) were added to DMEM at standard concentrations to avoid bacterial contamination (see materials and methods), as sera and mucosal fluids were not sterile. **(B)** SARS-CoV-2 neutralizing capacity of serum and mucosal samples was assessed by quantifying the inhibition of ACE2-RBD interaction using the cPass GenScript kit. The percentage of neutralizing capacity of samples is presented. Samples were not further titrated as their neutralizing capacity was low. Differences in ID_50_ titers or percentage of neutralization were analyzed applying ordinary one-way ANOVA followed by Dunnett´s multiple comparisons test (comparison with the negative control group). The mean from each group is indicated by a horizontal line. The serum graphic shows the percentage of neutralization values of the kit´s positive control and two convalescent sera, which were run in parallel with all the samples.

Once the dynamics of antigen-specific antibody responses were evaluated in the PRRSV-2 and SARS-CoV-2 experiments, we determined nAb responses in serum and mucosal samples collected at day 56. To evaluate the production of nAbs in animals from the PRRSV-2 experiment, MARC-145 cells and a PRRSV-2 VR-2332-derived isolate were employed. First, we confirmed that serum antibodies elicited by s3BT and BacDual-3BT recognized PRRSV-2 in fixed and permeabilized infected MARC-145 cells (**Supplementary Figure 9**). Next, we found that only serum samples from animals immunized with heat-inactivated PRRSV-2 or s3BT neutralized PRRSV-2 infection, compared to sera from mini-pigs immunized with the control baculovirus Bac-GFP (**Figure 6A**). The ID_50_ titers for PRRSV-2 and s3BT groups were 1:100 on average. None of the BacDual-3BT serum samples neutralized PRRSV-2 at statistically significant levels regarding negative control samples. In contrast, nasal mucus samples from the PRRSV-2 (*P* = 0.0108), s3BT (*P* = 0.010), and BacDual-3BT i.n. (*P* = <0.0001) groups neutralized PRRSV-2 infection, with ID_50_ titers close to 1:10 (**Figure 6A**). In saliva, no neutralizing activity was detected, while vaginal swab samples from the PRRSV-2 (*P* = 0.0005) and s3BT (*P* = 0.0223) groups neutralized PRRSV-2 when compared to the Bac-GFP control group (**Figure 6A** **)**. These results indicated that the soluble version of 3BT was more efficient than BacDual-3BT to induce nAbs in serum and vaginal fluid. In contrast, intranasally administered BacDual-3BT was a highly potent vector to elicit local nAbs against PRRSV-2.

To evaluate if NG19m-immunized animals produced nAbs in serum and mucosae, samples were tested using a commercial kit that measures the antibody-blocking activity of ACE2-RBD interaction. Only NG19m-subcutaneously immunized pigs produced nAbs in serum (*P* = 0.0158) in comparison to the adjuvant group (**Figure 6B**). Nasal mucus from animals belonging to the s.c.-i.n. (*P* = 0.0257) and i.n. (*P* = 0.0218) groups inhibited ACE2-RBD interaction when contrasted with the control group, as well as BAL samples from the same groups (*P* = <0.0001) (**Figure 6B**). Neutralizing activity in saliva was found in s.c.-i.n. group only (*P* = 0.0036) (**Figure 6B**). These results showed that NG19m induces more nAbs in serum when administered s.c., but when applied s.c.-i.n. or i.n., it stimulates a modest nAb response in the respiratory tract. These results may be improved by adjusting the doses of antigen and adjuvant applied in future experiments.

## Discussion

Effective mucosal vaccines have the potential to limit mucosal infection and inhibit pathogen transmission, representing an approach to deal with emerging and re-emerging viruses (14, 15, 89). Most licensed mucosal vaccines are currently based on live-attenuated whole-microorganisms, as those are the most immunogenic formulations to prevent infectious diseases (18, 90–93). Nevertheless, antigenically variable and highly transmissible pathogens such as PRRSV-2 and SARS-CoV-2 are not entirely suitable to be targeted by these immunogens, as a reversion to virulence may occur (94, 95). To avoid this problem, cutting-edge platforms and antigen-discovery approaches developed for parenteral vaccines might be tried for mucosal immunization, including computational epitope-based immunogen design (96, 97). This approach is an appealing method to focus immune responses on structural epitopes with proven or theoretical protective potential, removing the influence of immunodominant decoy epitopes and glycan shields (98, 99). However, using the resultant soluble proteins has the disadvantage of poor mucosal immunogenicity and the possible induction of immune tolerance (18). The data presented here demonstrate the production of binding and nAbs in animals immunized either *via* a combined s.c.-i.n. scheme with designed soluble epitope-based antigens or employing an intranasally delivered baculoviral vector. With any strategy, we avoided the administration of mucosal adjuvants and the induction of mucosal tolerance to the assessed antigens (100, 101).

Mucosal immunization against PRRSV-2 and SARS-CoV-2 has already been reported utilizing different platforms (14, 89, 102). For PRRSV-2, intranasal and parenteral live-attenuated vaccines have been evaluated, with the risk of mucosal shedding and reversion to virulence (95, 103, 104). Besides, experimental mucosal immunogens based on recombinant proteins, VLPs, or viral vectors have targeted PRRSV-2 surface glycoproteins, with the disadvantage of including decoy epitopes and glycan shields (30, 32). Our rationale in choosing the conserved epitope B to design 3BT was based on studies in which peptide-binding and phage-display assays demonstrated that infection-elicited nAbs bind to epitope B in PRRSV-2 (75, 77, 105). Another reason was the documented participation of the GP5 ectodomain in virus attachment and internalization into porcine macrophages, aided by N-glycans localized in epitope B, as it interacts with heparan sulfate proteoglycans (106), sialoadhesin (107, 108), and the non-muscle myosin heavy chain 9 (MYH9) (109, 110). In the case of SARS-CoV-2, RBD binds to angiotensin-converting enzyme 2 (ACE2) to enter cell cytoplasm, representing a key target for nAbs (111, 112). Different RBD-specific cross-reactive monoclonal antibodies that neutralize SARS-CoV and SARS-CoV-2 have been isolated from convalescent sera, which implies that RBD could be used in pan-sarbecovirus vaccines (113, 114).

In this work, we observed that antigen formulation (soluble or vectored), the immunization route (s.c., i.n. or combined), and the adjuvant employed for parenteral immunization determined the magnitude of serum and mucosal humoral immune responses. In the PRRSV-2 experiment, s3BT elicited a higher concentration of antigen-binding antibodies in serum, saliva, and vaginal swab samples than BacDual-3BT. Two factors might partially explain these results: the adjuvant used for parenteral inoculation of the soluble protein and the adaptive immune responses elicited against the baculoviral vector.

Earlier, our group demonstrated that complete and incomplete Freund´s adjuvants (CFA and IFA) stimulate antigen-specific humoral immune responses when mini-pigs are subcutaneously primed and boosted with an inactivated virus (86). As CFA is currently prohibited, here we utilized IFA only. IFA is a classical water-in-oil (W/O) emulsion system made of nonmetabolizable oils, favoring the production of Th2-biased cytokine responses and high antibody titers (115, 116). Despite IFA lacks the mycobacterial component and causes less granuloma formation and pain than CFA, its use in preclinical studies has been replaced by less toxic W/O adjuvants, such as Montanide ISA™ 51 and 720 (117). Besides, newer squalene-based oil-in-water emulsified adjuvants, such as AS03 and MF59, are used in licensed human vaccines, as they are more readily metabolizable, less toxic, and better characterized (118–120). Then, a perspective in our laboratory is to explore the serum and mucosal antibody responses against recombinant proteins, using the newer adjuvants already mentioned and the combined s.c.-i.n. immunization scheme.

The lower concentrations of systemic, salival, and vaginal antibodies induced by BacDual-3BT might indicate that the total amount of 3BT antigen delivered by the vector (1 x 10^8^ PFU) was inferior to the inoculated quantity of s3BT protein. Very likely, the systemic anti-baculovirus immune responses generated after the first s.c. dose impaired its transduction capacity in subsequent immunizations. In this regard, various studies have shown the high immunogenicity of the baculoviral vector AcMNPV (121, 122). The innate sensing of CpG-DNA motifs in the baculoviral DNA, similar in abundance to those from *E. coli* and herpes simplex virus, results in the secretion of pro-inflammatory cytokines and type I IFNs (123). Then, cellular and humoral immune responses are produced against both the antigen of interest and the vector itself, reducing its subsequent transduction capability of host cells (28, 124). More research is needed to assess the advantages and limitations of the baculovirus AcMNPV as a mucosal immunization vector (32).

In contrast with results in serum, saliva, and vaginal swabs, animals immunized with BacDual-3BT only *via* the intranasal route produced an elevated concentration of 3BT-binding IgA in nasal mucus, comparable to the s3BT group. Hypothetically, when BacDual-3BT was intranasally instilled, the IgA response was better focused on the surface 3BT-GS antigen than on capsid and other structural baculoviral proteins. For instance, mice mucosally exposed to bacteria develop a surface antigen-focused IgA repertoire, in contrast to systemic deliverance, which results in a broad IgG response directed to both cytoplasmic and cell-surface bacterial antigens (125). In concordance with binding-IgA found in nasal mucus, intranasally delivered BacDual-3BT elicited similar titers of PRRSV-2 neutralizing IgA compared to s3BT. These results agree with reports in which intranasal vaccination with recombinant baculoviruses elicited local Abs against several mucosal pathogens, such as influenza and human papillomavirus (126, 127).

Only s3BT elicited anti-PRRSV-2 nAbs in sera, with average titers of 1:100. In agreement with such finding, a recent report demonstrated that VLPs displaying epitope B induced serum nAbs, although titers were below those from animals immunized with a PRRSV-2 attenuated-live vaccine (128). Similarly, in another work, a mosaic DNA vaccine that included epitopes B and T2 provided superior protection against heterologous challenge than a GP5 DNA control vaccine (52). In this sense, serum nAbs protect pigs from homologous PRRSV-2 infection (129). In passive-transfer experiments, it was determined that the minimum serum nAb titer to avoid viremia in PRRSV-2-challenged pigs was 1:8 (130). In contrast, a titer of 1:32 was useful to generate sterilizing immunity in 50% of animals. Although no viremia was detected in the blood of animals that received nAbs (1:32), they transmitted the virus during the last 5 of 14 days post-infection, suggesting that locally produced nasal and salival antibodies are also needed (130). In this regard, dimeric and monomeric salival IgA isolated from farm animal samples reduced PRRSV-2 macrophage infection *in vitro* more efficiently than IgG (131).

Regarding NG19m, the trend observed was weak humoral immune responses, irrespective of the inoculation route. Serum nAbs were detected in samples from the s.c. group only, and a low neutralization activity was found in nasal mucus, saliva, and BAL samples from the s.c.-i.n. and i.n. groups. Reports demonstrated that soluble monomeric RBD, although more affordable and straightforward to be produced, is less immunogenic than multimeric RBD versions (132). Such reduced immunogenicity is explained by the lesser capacity of monomeric antigens to induce BCR crosslinking and the consequent poor B-cell activation (133, 134). Therefore, new strategies have been developed to enhance RBD immunogenicity, such as the formulation of multivalent RBD nanoparticles, VLPs, or RBD-carrier protein conjugate vaccines (133, 135). Furthermore, alum employed with NG19m, although widely used in approved subunit vaccines, promotes weaker humoral immune responses to soluble proteins in serum and mucosae compared to IFA or new adjuvant formulations (134, 136). Despite this, the scarce nAbs induced by NG19m in mucosae might be protective *in vivo*, as mucosal dimeric IgA has a neutralization potency 7.5-fold higher than serum IgG against SARS-CoV-2 (23, 137).

Overall, our results indicate the feasibility of using computationally designed antigens and a combined s.c.-i.n. immunization scheme to generate systemic and mucosal antibodies against emerging and re-emerging viruses. However, this study has limitations. Although we have proven that designed epitope-based antigens induce binding and nAbs in serum and mucosae, we do not know if such humoral immune responses protect from infection. In the case of PRRSV-2, our results set the ground for future challenge experiments, which could indicate if serum and mucosal nAbs induced by s3BT and BacDual-3BT protect against mucosal and systemic infection with homologous and, most importantly, heterologous PRRSV-2 strains. For NG19m targeting SARS-CoV-2, mini-pigs are a suitable model to evaluate the mucosal immune responses generated, given their body size and anatomic similitudes to humans (138); nevertheless, they are not susceptible to SARS-CoV-2 infection. Transgenic mice expressing ACE2 or non-human primates are the appropriate experimental animal models to perform challenge experiments (139). Concerning adjuvants used, alum was not immunogenic enough and, as already discussed, IFA is not approved for human or veterinary employment, so newer adjuvants should be further explored for s.c. priming (140). Moreover, the baculovirus BacDual-3BT was a good intranasal immunogen but a poor systemic vaccine vector, so we should further evaluate a combination of soluble and vectored antigens. Finally, we need to analyze the produced resident T and B cell memory cell populations and the durability of the systemic and mucosal humoral immune responses after mucosal immunization.

## Data Availability Statement

The original contributions presented in the study are included in the article/**Supplementary Material**. Further inquiries can be directed to the corresponding author.

## Ethics Statement

The animal study was reviewed and approved by Institutional Animal Care and Use Committee, Center for Research and Advanced Studies (CINVESTAV).

## Author Contributions

Study design, MF-S, RA-M, BX-C, and MV-L. Soluble 3BT design and bioinformatic analyses, MF-S. Soluble 3BT and NG19m production, MF-S, LN-M, GM-P, MS-C, RG-G, RR-M, and BX-C. BacDual-3BT production and characterization, MF-S, CR-E, AG-V, SR-M, and VR-A. Immunization, sample collection, and processing, MF-S, CR-E, DP-G, JR-F, and GT-F. Data analyses and manuscript first draft writing, MF-S. Data analyses and manuscript editing, MF-S, RA-M, BX-C, and MV-L. Funding acquisition, MF-S, RA-M, BX-C, and MV-L. All authors contributed to the article and approved the submitted version.

## Funding

This study was funded by Consejo Nacional de Ciencia y Tecnología (CONACYT), grant no. 2015-01-235, Asociación IPVS Mexico 2014 A.C., grant to the project: Mucosal vaccination using a multiple antigen-expressing recombinant baculovirus platform, The porcine reproductive and respiratory syndrome (PRRS) as a model of infectious emerging and reemerging diseases, and Agencia Mexicana de Cooperación para el Desarrollo (AMEXCID), grant no. AMEXCID 2020/7. MF-S received a doctoral scholarship from CONACYT (no. 449596).

## Conflict of Interest

The authors declare that the research was conducted in the absence of any commercial or financial relationships that could be construed as a potential conflict of interest.

## Publisher’s Note

All claims expressed in this article are solely those of the authors and do not necessarily represent those of their affiliated organizations, or those of the publisher, the editors and the reviewers. Any product that may be evaluated in this article, or claim that may be made by its manufacturer, is not guaranteed or endorsed by the publisher.
