# Supplementary material for: Combined Subcutaneous-Intranasal Immunization With Epitope-Based Antigens Elicits Binding and Neutralizing Antibody Responses in Serum and Mucosae Against PRRSV-2 and SARS-CoV-2
Source: Front Immunol. 2022 Mar 31;13:848054. doi: 10.3389/fimmu.2022.848054 (PMC9008747; doi:10.3389/fimmu.2022.848054)
Supplement: Supplementary file 1 [file DataSheet_1.docx]

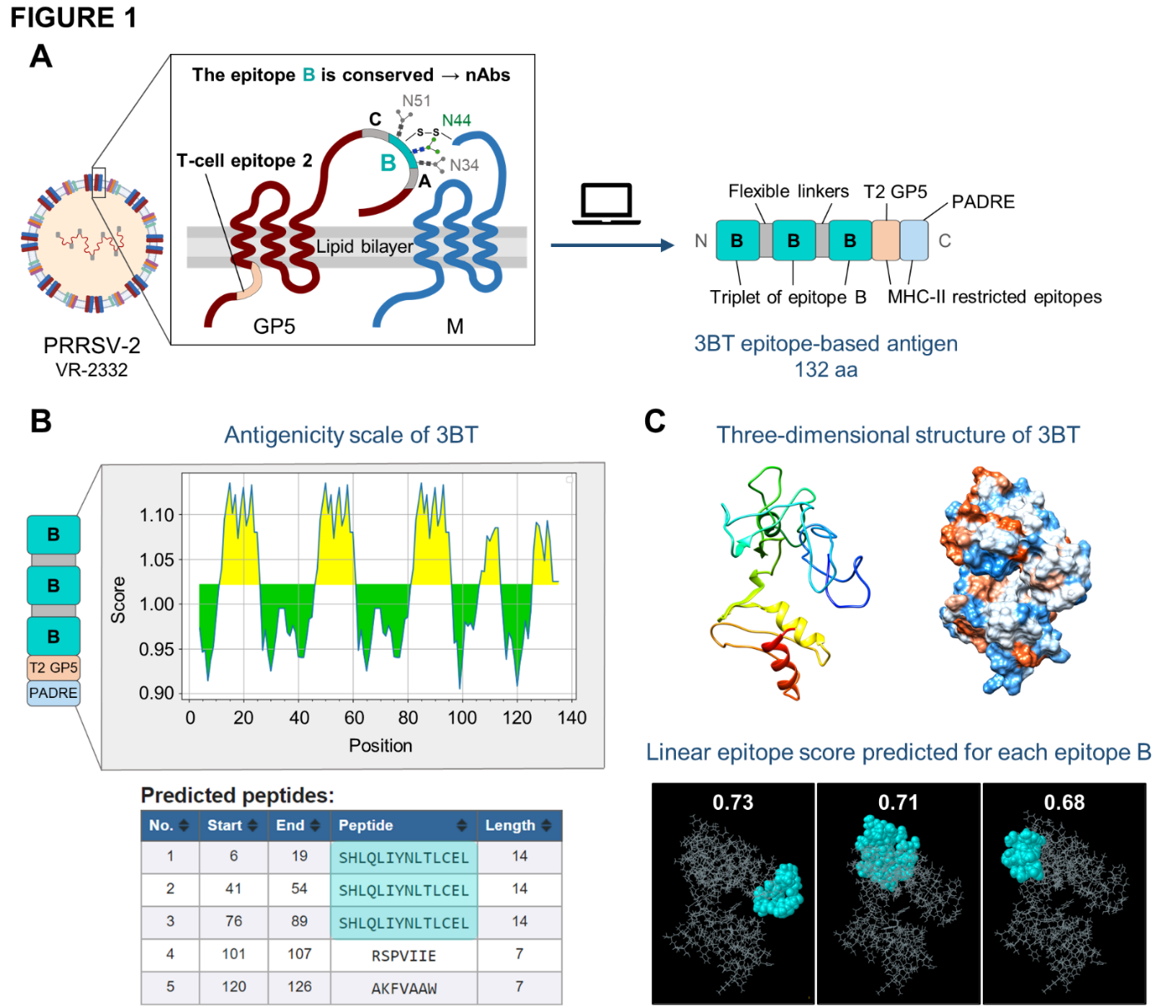


**SUPPLEMENTARY FIGURE 1** │Design and bioinformatic analysis of the 3BT epitope-based antigen. **(A)** The DNA sequence coding for the 3BT epitope-based antigen was designed from epitopes B and T2 of the GP5 protein of the PRRSV-2, VR-2332 reference strain. The 3BT epitope-based antigen contains a triplet of epitope B in tandem, two flexible linkers in between, and the MHC-II-restricted epitopes T2 and PADRE. PADRE, Pan-DR T helper epitope. **(B)** Kolaskar & Tongaonkar antigenicity scale of 3BT, showing the results obtained from the Immune Epitope Database (IEDB). The first three most antigenic predicted peptides listed in the table include each repeat of the core of the epitope B (highlighted in cyan). **(C)** The three-dimensional structure of 3BT was modeled using I-TASSER, refined through GalaxyRefine 2 server, and visualized in UCSF Chimera. The linear epitope score predicted for each epitope B in the three-dimensional structure of 3BT was evaluated with the ElliPro tool in IEDB. Figure partially created with BioRender.com.

N

C


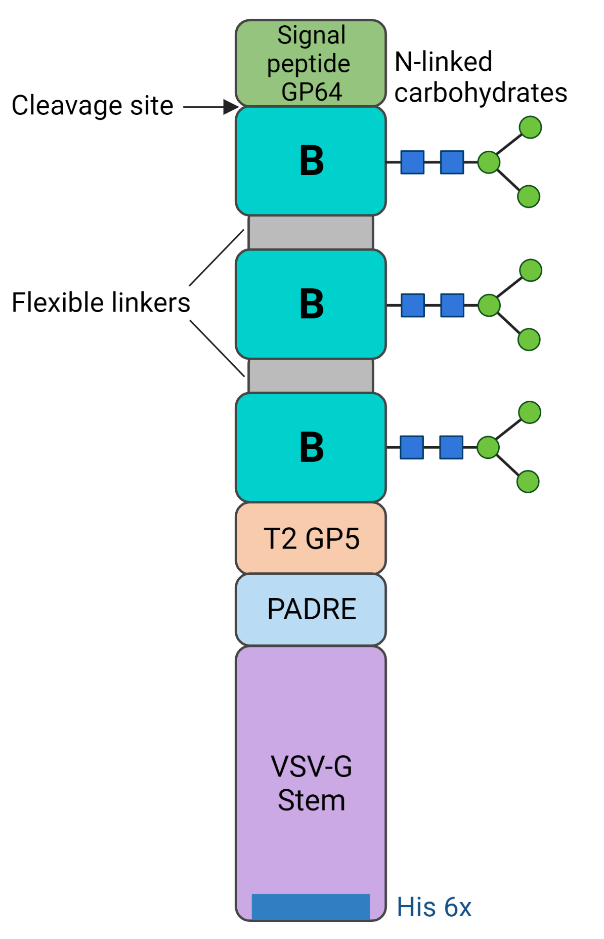


**A**

**B**

MVSAIVLYVLLAAAAHSAFA*AELETR*SGDSSSHLQLIYNLTLCELSGTDGSAGSAAGSGEFSGDSSSHLQLIYNLTLCELSGTDGSAGSAAGSGEFSGDSSSHLQLIYNLTLCELSGTDKGRLYRWRSPVIIEKAA*TRGARTEFS*AKFVAAWTLKAAAFEHPHIQDAASQLPDDESLFFGDTGLSKNPIELVEGWFSSWKSSIASFFFIIGLIIGLFLVLRVGIHLCIKLKHTKKRQIYTDIEMNRLGKHHHHHH

**SUPPLEMENTARY FIGURE 2 │** The amino acid sequence of the 3BT-GS protein. **(A)** Schematic representation of the 3BT-GS protein, designed to be expressed in baculovirus. The signal peptide from GP64, included at the N-terminal end of 3BT-GS, is cleaved in the endoplasmic reticulum of eukaryotic cells, especially in insect Sf9 cells. **(B)** The complete sequence of the 3BT-GS protein is shown (255 aa), with its features indicated by the corresponding color. Repeats of epitope B and T-cell epitopes T2 and PADRE are underlined. Pale gray amino acid residues, italicized, come from restriction sites included in the *64-3bt-gs* synthetic gene. Figure partially created with BioRender.com.


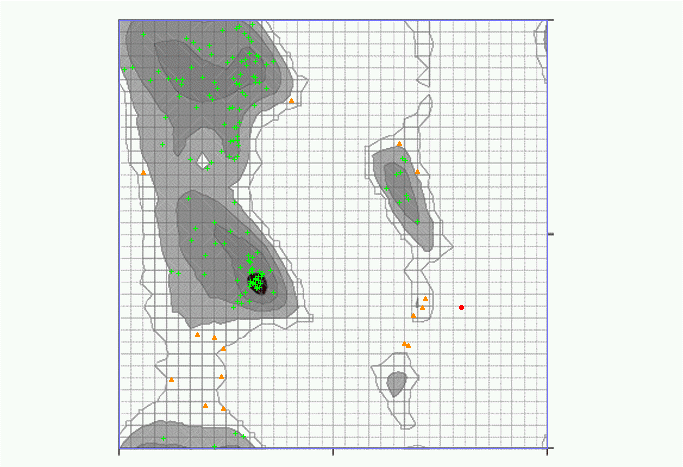


**SUPPLEMENTARY FIGURE 3 │** Ramachandran plot for validation of the predicted tridimensional structure of 3BT. The plot was obtained from Ramachandran Plot Server (see materials and methods). Green, highly preferred observations (138 = 89.03%); orange, preferred observations (16 = 10.32%); red, questionable observations (1 = 0.64%).


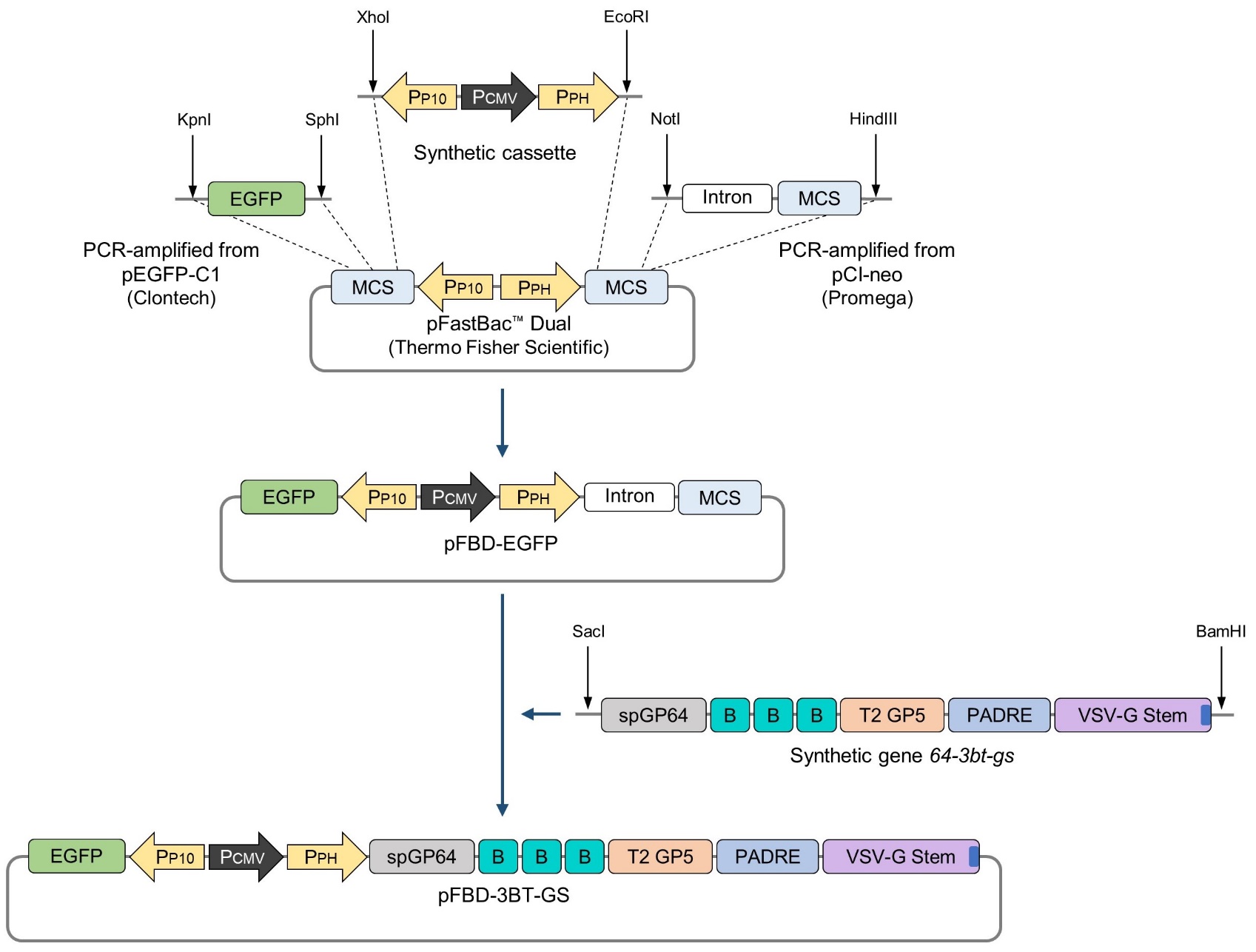


**SUPPLEMENTARY FIGURE 4 │** Molecular cloning process to obtain the pFBD-3BT-GS baculovirus-expression vector.

PNGase F

- +

kDa

180

100

70

55

40

35

25

BacDual-3BT

Anti His6x


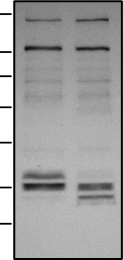


**A**

**B**


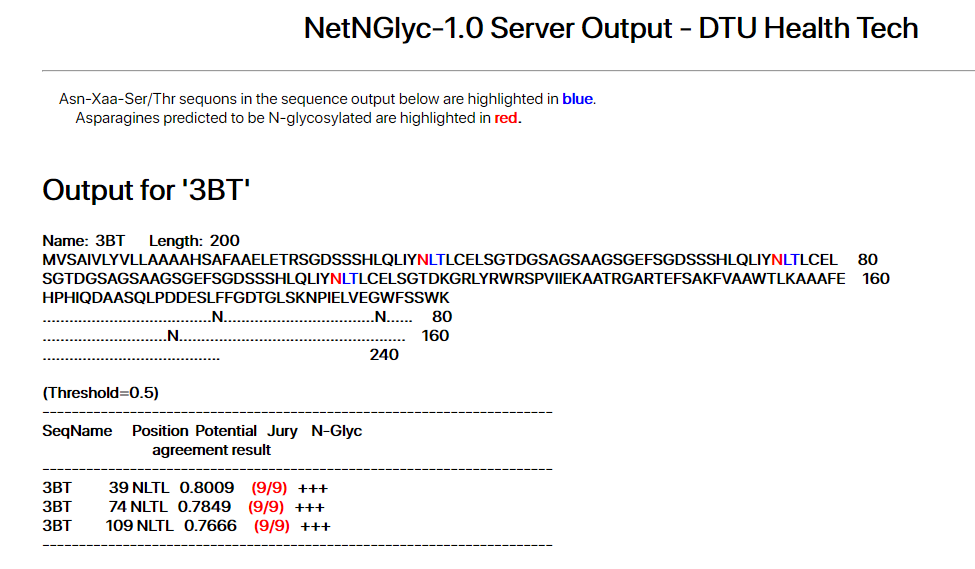


**SUPPLEMENTARY FIGURE 5 │**The 3BT-GS protein displayed on the membrane of BacDual-3BT is glycosylated. **(A)** Calculated glycosylation potential of Asn residues in the core of each copy of epitope B in 3BT. **(B)** Concentrated virions of BacDual-3BT were treated with PNGase F and examined through immunoblot, using a primary anti-His6x antibody.

Anti-PRRSV-2 convalescent swine serum 1

BacDual-3BT

PRRSV-2

Bac-GFP

kDa

180

100

70

55

40

35

25

3BT-GS

GP5

M


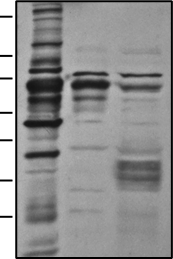

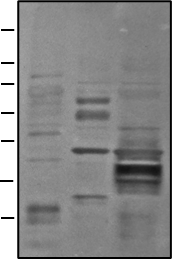


Anti-PRRSV-2 convalescent swine serum 2

BacDual-3BT

PRRSV-2

Bac-GFP

kDa

180

100

70

55

40

35

25

3BT-GS

GP5

M

**SUPPLEMENTARY FIGURE 6 │**The 3BT-GS protein displayed on the membrane of BacDual-3BT is recognized by anti-PRRSV-2 convalescent swine sera. Immunoblot of virions of BacDual-3BT incubated with anti-PRRSV-2 convalescent swine sera. Concentrated virions of PRRSV-2 and Bac-GFP were the positive and negative control, respectively. Antibodies from convalescent sera recognize the GP5 and M proteins in PRRSV-2.


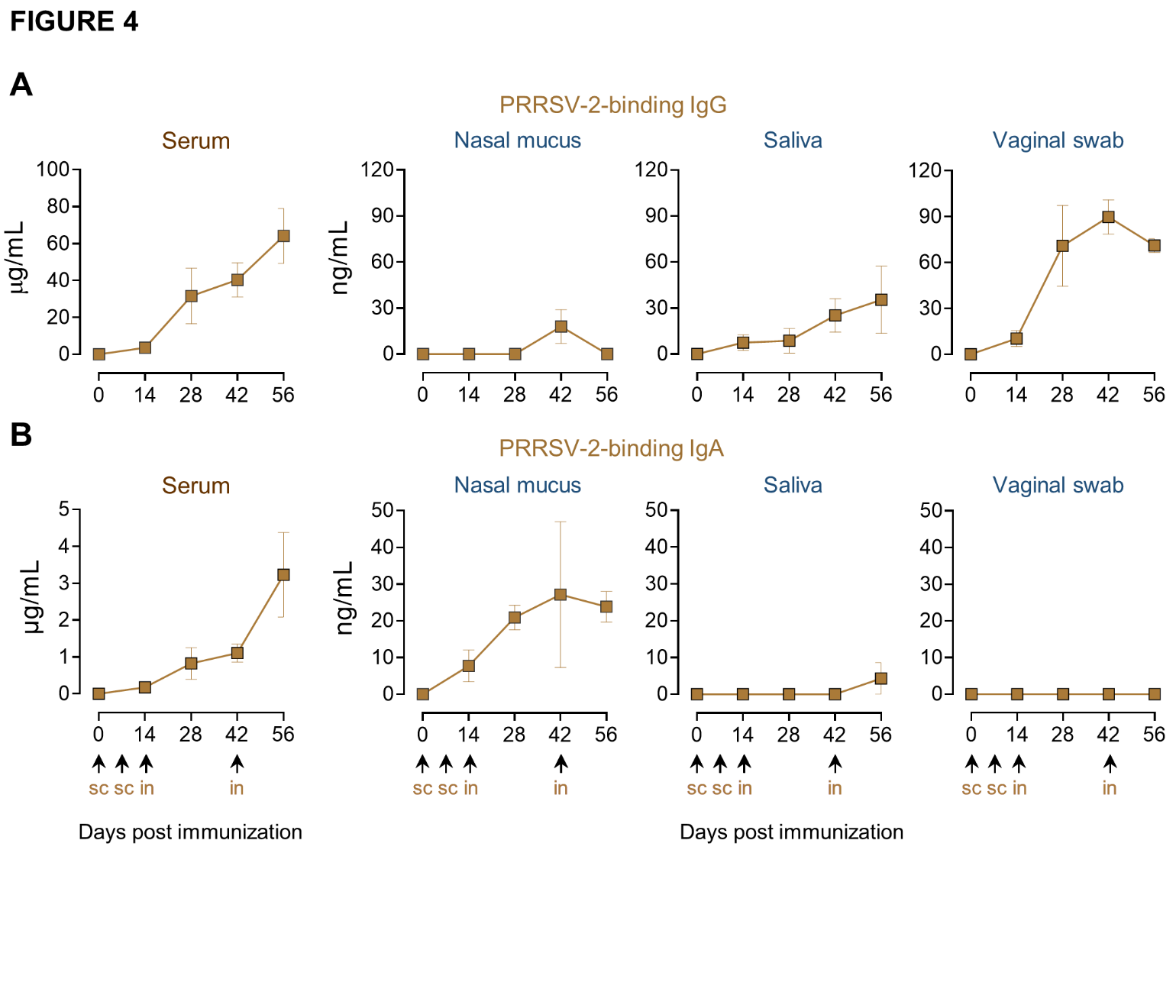


**SUPPLEMENTARY FIGURE 7** │Subcutaneous-intranasal immunization with heat-inactivated PRRSV-2 drives the production of antigen-binding IgG and IgA in serum and mucosal fluids. Animals received 100 µg of heat-inactivated PRRSV-2 mixed with IFA 1:1 (v/v) in the first two s.c. doses, and 200 µg of PRRSV-2 without adjuvant for intranasal immunizations. Kinetics of serum and mucosal anti-PRRSV-2 IgG **(A)** and IgA **(B)**, assayed by quantitative ELISA using heat-inactivated PRRSV-2. Error bars represent SEM.

**A**


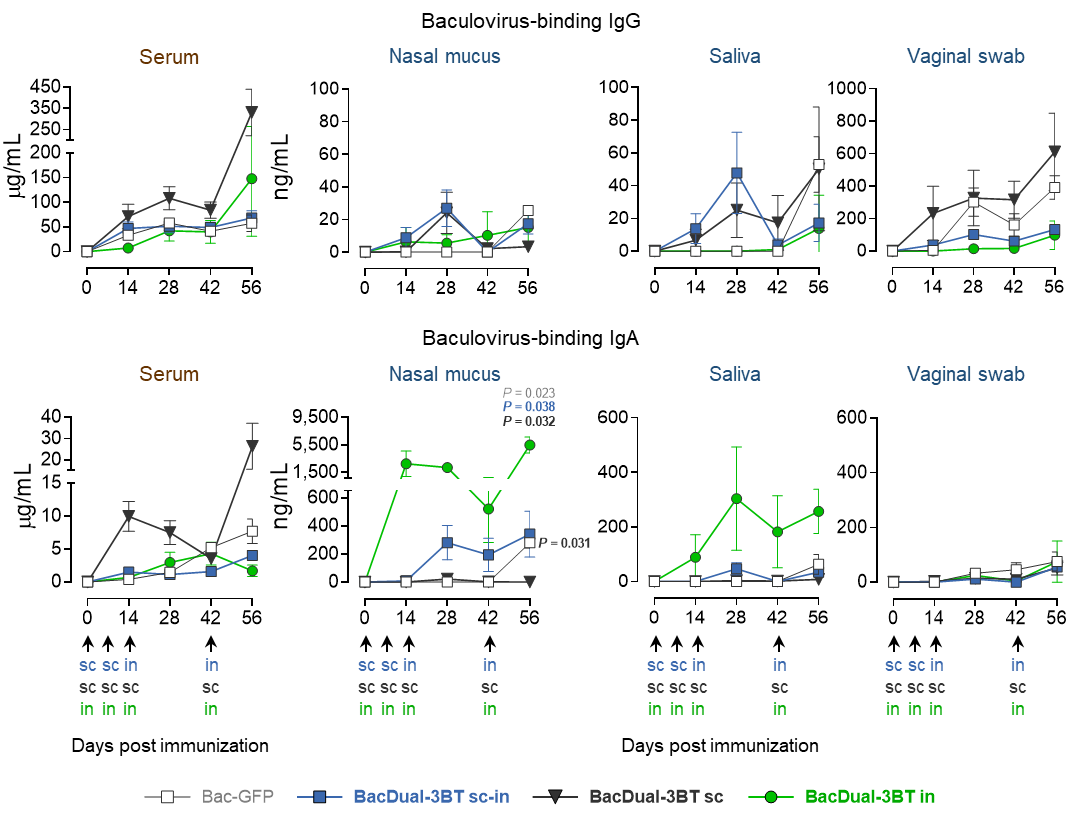


**B**

**SUPPLEMENTARY FIGURE 8 │** Comparative dynamics of serum and mucosal baculovirus-binding IgG and IgA in animals immunized with recombinant baculoviruses. Mini pigs were vaccinated with 5 x 10^8^ PFU of recombinant baculoviruses in the first two doses, regardless of the route, and 1 x 10^9^ PFU in the last two doses. Kinetics of serum and mucosal baculovirus-binding IgG **(A)** and IgA **(B)**, assayed by quantitative ELISA using concentrated virions of Bac-GFP. Differences in the levels of antibodies among the groups were analyzed utilizing two-way ANOVA with the Geisser-Greenhouse correction, followed by Dunnett´s (comparison with control group) and Tukey´s (comparison among the groups) multiple comparisons. Error bars represent SEM. P values ≤ 0.05 are indicated for day 56 only.

**SUPPLEMENTARY FIGURE 9 │**Serum IgG from s3BT and BacDual-3BT-immunized mini pigs binds PRRSV-2. The ability of serum IgG to recognize PRRSV-2 was measured via in-cell ELISA. MARC-145 cells grown in 96-well plates were infected with PRRSV-2 for 48 h. Cells were fixed, permeabilized, and incubated with serum samples collected at day 56, and serial twofold dilutions were obtained. A goat anti-pig IgG-HRP secondary Ab was utilized, and after adding TMB, absorbance was measured at 450 nm. Error bars represent SEM.

**Supplementary Table 1** │Theoretical physicochemical properties of s3BT and 3BT-GS.

| **Property** | **s3BT** | **3BT-GS** |
| --- | --- | --- |
| Length (aa) | 188 | 235 |
| Molecular weight (kDa) | 19.9 | 25.5 (without N-linked carbohydrates)  ~35-36 (with mannose and paucimannose N-glycans) |
| Positive charged residues | 11 | 20 |
| Negative charged residues | 22 | 25 |
| Isoelectric point | 5.42 | 6.59 |
| Charge at pH 7.0 | -9.86 | -3.90 |
| Grand average of hydropathicity (GRAVY) | -0.319 (hydrophilic) | -0.118 (hydrophilic) |
| Instability index | 38.78 (stable) | 38.03 (stable) |

**Supplementary Table 2** │Primers utilized to obtain the expression vectors pFBD-3BT-GS and pCri-8a-3BT.

| **Primer name** | **Sequence (5´- 3´)** |
| --- | --- |
| EGFP-SphI_F | GTCATAGCATGCTTCAGATCCGCTAGCGCTAC |
| EGFP-KpnI_R | CAGGTACCTAATCATTAGCTGAGTCCGGACTTGTACAG |
| NeoInt-NotI_F | TGCAGCGGCCGCCTCGTTTAGTGAACCGTCAG |
| Neo-HindIII_R | GTCATAAAGCTTTCGAGGCTAGCCTATAGTG |
| NcoI-Ecto_F | CATGCCATGGCGGAACTCGAGACGCGTAGC |
| HindIII-Ecto-R | CCCAAGCTTTCATTTGGATAGCCCAGTATCACCA |
